# The short-term effects of air pollution on respiratory disease hospitalizations in 5 cities in Poland: comparison of time-series and case-crossover analyses

**DOI:** 10.1007/s11356-020-08542-5

**Published:** 2020-04-30

**Authors:** Alessandro Slama, Andrzej Śliwczyński, Jolanta Woźnica-Pyzikiewicz, Maciej Zdrolik, Bartłomiej Wiśnicki, Jakub Kubajek, Olga Turżańska-Wieczorek, Marcin Studnicki, Waldemar Wierzba, Edward Franek

**Affiliations:** 1 Central Clinical Hospital MSWiA in Warsaw, Wołoska 137, 02-507 Warsaw, Poland; 2 University of Humanities and Economics in Łodz, Satellite Campus in Warsaw, ul. Wolność 2a, 01-018 Warsaw, Poland; 3 Chancellery of the Prime Minister of Poland, al. Ujazdowskie 1/3, 00-001 Warsaw, Poland; 4 Department of Business Economics, Warsaw School of Economics, Al. Niepodleglosci 162, 02-554 Warsaw, Poland; 5 Warsaw University of Life Sciences, Nowoursynowska 166, 02-787 Warsaw, Poland; 6 Mossakowski Clinical Research Centre, Polish Academy of Sciences, Pawinskiego 5, 02-106 Warsaw, Poland

**Keywords:** Hospitalizations, Air-pollution, Case-crossover, Time series, Lag effect, Respiratory diseases

## Abstract

Very few publications have compared different study designs investigating the short-term effects of air pollutants on healthcare visits and hospitalizations for respiratory tract diseases. This study describes, using two different study designs (a case-crossover design and a time-series analysis), the association of air pollutants and respiratory disease hospitalizations. The study has been conducted on 5 cities in Poland on a timeline of almost 4 years. DLNM and regression models were both used for the assessment of the short-term effects of air pollution peaks on respiratory hospitalizations. Both case-crossover and time-series studies equally revealed a positive association between air pollution peaks and hospitalization occurrences. Results were provided in the form of percentage increase of a respiratory visit/hospitalization, for each 10-μg/m^3^ increment in single pollutant level for both study designs. The most significant estimated % increases of hospitalizations linked to increase of 10 μg/m^3^ of pollutant have been recorded in general with particulate matter, with highest values for 24 h PM_2.5_ in Warsaw (6.4%, case-crossover; 4.5%, time series, respectively) and in Białystok (5.6%, case-crossover; 4.5%, time series, respectively). The case-crossover analysis results have shown a larger CI in comparison to the results of the time-series analysis, while the lag days were easier to identify with the case-crossover design. The trends and the overlap of the results occurring from both methods are good and show applicability of both study designs to air pollution effects on short-term hospitalizations.

## Introduction

Atmospheric pollution is representing globally the highest environmental risk for the human health. The WHO has published an estimation showing that 4.2 million deaths every year as a result of exposure to ambient (outdoor) air pollution and 3.8 million deaths every year as a result of household exposure to smoke from dirty cookstoves and fuels (WHO 2020). Large longitudinal studies (Fisher et al. 2015) were capable of demonstrating that the exposure over a long period of time to air pollutants can be linked to nonaccidental and cause-specific mortality. A recent catalog and synthesis review of the relationships of air pollution with long-term morbidity (Lipfert 2018) has listed 489 publications which covered respiratory, cardiovascular, cancer, neurological, diabetic, and birth outcomes in relation to 12 air pollutants. In approximately 50% of these publications, the relationships that have been found were statistically significant. Also short time exposure to air pollution can cause health effects, e.g., it may trigger an asthma attack (Noh et al. 2016) or worsen a preexisting medical condition. Short-term effects of pollution may trigger or exacerbate the diseases of the eye and adnexa (Song et al. 2019), respiratory diseases (Xie et al. 2019), and cardiovascular diseases (Ma et al. 2017) and may also cause premature death (Dastoorpoor et al. 2018). “The time series, case-crossover, and panel studies are best suited for estimating the acute effects of air pollution” (Peng and Dominici 2008). When it comes to evaluating hospitalizations or healthcare visits or mortality as outcomes of a given study, the data structure and the type of effects that need to be studied typically lead to the model selection. Often the choice falls on regression models. Both time-series (Bell et al. 2004; Zheng et al. 2015; Jie et al. 2018) and case-crossover designs are widely used (Taj et al. 2017; Leiser et al. 2018). Although, in addition to these consolidated study designs, for the same purposes, also other methods are being routinely used, e.g., cross-sectional studies or case-control studies (Nieuwenhuijsen 2015). In any case, time-series analyses are commonly used to investigate the association of pollution exposures (which vary in time) with the investigated event counts (e.g., hospitalizations), while the case-crossover design was originally designed to study “acute transient effects of intermittent exposures” (Maclure 1991). While originally most of such studies (Fung et al. 2003; Hajat 2003) used time-series analyses, progressively case-crossover studies have become more and more the alternative analytical approach (Duan et al. 2016). Both techniques have their own advantages and drawbacks. Only very few studies have compared the output of the two methodologies (Lin et al. 2002; Yuming et al. 2010; Kayo et al. 2009; Tong et al. 2012; Meng et al. 2017; Zheng et al. 2015), in particular on the respiratory hospitalizations and healthcare visits associated with air pollution. This paper compares the already published results of the impact of air pollution on hospital admissions with a focus on respiratory diseases performed with a time-series multicity analysis (Slama et al. 2019) with a case-crossover design performed on the same dataset.

## Methods

**Fig. 1:**
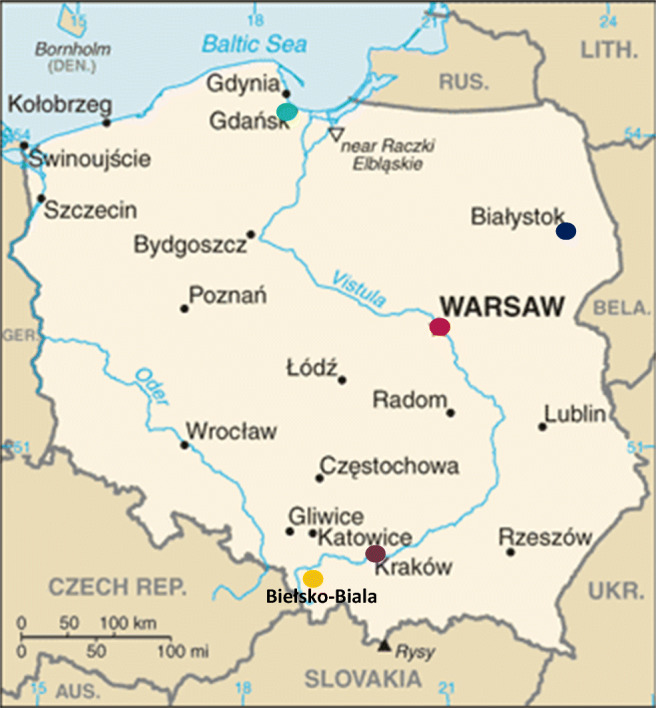
Map of Poland* and the cities chosen in both study design analysis. *The map is taken from the CIA World Factbook

The detailed description of the data sources, cities, health data, and pollutants data have been published previously (Slama et al. 2019). In brief, five cities in Poland have been chosen to provide the appropriate representation of the country data. Two large cities (Warsaw 2 M inhabitants and Krakow 0,8 M), one medium (Gdansk 0,5 M) and two smaller (Bielsko-Biała and Białystok 0,3 M); in total 3,9 M – approximately 10% of the total population of Poland, have been selected to represent different city-size demographics. In addition, historical data show different pollution levels, with Krakow, Warsaw, and Bielsko-Biała being significantly more polluted than the other cities. As a last point, their climatic and geographical location (costal Gdansk, continental Warsaw, Gdansk north, Krakow and Bielsko-Biała South, Białystok East) as well as availability of data, drove the ultimate choice. A map of the study area is reported in Fig. 1. The data related to the number of hospitalizations in the cities of Warsaw, Białystok, Bielsko-Biała, Krakow, and Gdansk were obtained from the reporting system of the NHF (in Polish: Narodowy Fundusz Zdrowia) and covered a period of almost 4 years (2014–2017, 1255 days). Data on the concentration of air pollution were obtained from the Chief Inspectorate for Environmental Protection (GIOS) and included NO, NO_x_, NO_2_, O_3_, SO_2_, PM_2.5_, PM_10_, PM_10_24_, and PM_2.5_24_. Daily (obtained from manual stations) and hourly data (obtained from automatic stations, coded as 24, e.g., PM_10_24_, for which the 24 h average have been recorded) have been used in the analysis. Meteorological data have been gathered from the Institute of Meteorology and Water Management (IMGW) that have beacons in the Polish cities and included temperature, main wind speed, and precipitations.

### Statistical analysis

The study has been designed to compare the results of two different study designs (time series and case-crossover).

- In the original, already published time series analysis (Slama et al. 2019), the hypothesized association between air pollution and respiratory hospitalizations has been analyzed using a simple correlation analysis, later combined with a DLNM model based on Almon method, to explore the lag effect. Normalization of the sample for factors like temperature and day of the week had to be put in place to adjust for seasonality and time dependent trends.

b. The case-crossover study design is per se designed to adjust for seasonality trends and time (Perrakis et al. 2014), so no normalization for temperature and/or day of the week had to be put in place. A “Gasparrini”-type (Gasparrini et al. 2010, 2012; Gasparrini 2011) DLNM model has been used to explore the association between air pollution and respiratory hospitalization with respect to the potential time delay, the so-called time lag. The lag assumption in the analysis was that the effect of increased air pollution may have a lag effect from 0,1,2… up to 10 days. The decision to stop the analysis at day 10 has been taken after analysis of the relevant literature, which insofar reported lag effects of few days only for short-term respiratory effects. In the study model, smooth temperature functions were included, as well as wind speed and precipitation. Natural cubic splines with three degrees of freedom were used for both the nonlinear weather factors effect and the lagged effect. The month, year, and national holidays as non-overlapping strata helped to avoid bias due to overlap. In addition, the day of the week was matched with control days. The analysis has been carried out utilizing the statistical software R version 3.5.1 with “dlnm” and “splines” package.

Like for the already published results utilizing a time-series analysis, all estimates have been displayed as percentage increase in the number of respiratory disease hospitalizations, associated with a 10-μg/m^3^ increase of the air pollution level.

## Results

**Fig. 2:**
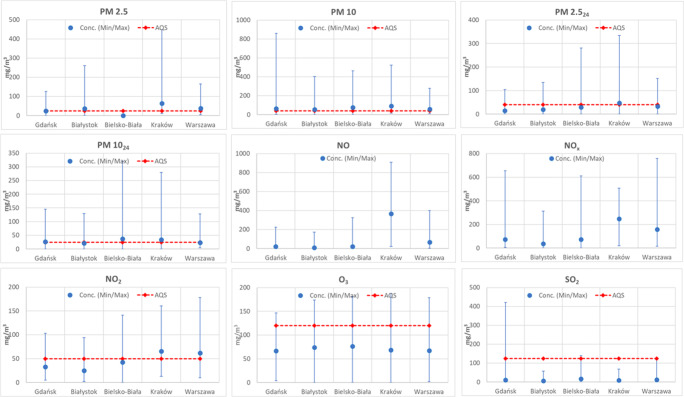
Comparison of the air pollution mean concentrations, their range (±), and the corresponding EU Air Quality Standards

The summary of the air pollution data and the main meteorological data throughout the study period are displayed in graphical format in Fig. 2. For a more thorough review of these data, we revert to the published data referred to in the introduction. In general, the cities show a pollution level which, especially for the particulate matter and in some cases (Warsaw and Krakow) for NO_2_, is often higher than the EU limits.

The results utilizing both the time-series analysis and the case-crossover design are displayed in Table 1, results which include the confidence interval (CI). The results are displayed in the form of expected percentage increase in hospitalizations per 10 unit increase in concentration of the air pollutant. The most significant increases in hospitalizations for respiratory disease have been identified (by both methods) in PM2.5_24 (Warsaw, 6.4 and 4.5, respectively, for time series (first value) and case-crossover (second value), and Białystok, 5.6 and 4.5, respectively), for SO_2_ (Krakow, 4.8 and 7.5, respectively, and Biełsko-Biala, 5.5 and 5.4, respectively), and for PM2.5 (Gdansk, 4.0 and 3.1, respectively).

**Table 1:** Comparison of percentage increase in hospitalization per each 10 units increase in pollutant concentration for both time-series and case-crossover study designs

	Pollutant	Białystok				Bielsko-Biała				Gdańsk				Kraków				Warszawa			
		%	CI (95%)		Lag	%	CI (95%)		Lag	%	CI (95%)		Lag	%	CI (95%)		Lag	%	CI (95%)		Lag
Case-crossover (Gasparrini)	PM2.5 (μg/m^3^)	1.72	0.92	2.54	1–3 and 9*	A				4.02	2.86	5.2	1–3	0.72	0.33	1.12	1–2	4.7	3.76	5.66	7–8
	PM10 (μg/m^3^)	0.68	0.2	1.16	1–3 and 9*	1.01	0.69	1.33	1–2	2.2	0.56	4.63	1–2	0.62	0.29	0.96	1–2	2.18	1.59	2.78	5–7
	PM2.5_24 (μg/m^3^)	5.61	4.19	7.05	1–3 and 9*	0.52	− 0.17	1,21	3–4	2.61	1,28	3,96	2–3	0.08	− 0.55	0.71	1–3	6.39	5.14	7.65	6–7
	PM10_24 (μg/m^3^)	2.11	0.83	3.4	1–3 and 9*	0,67	0,1	1,24	3–5	2,65	1,64	3,66	2–3	0,48	-0,05	1,01	1–3	4,6	3,54	5,67	6–7
	NO_x_ (μg/m^3^)	1,65	1,51	1,8	2–4	1.09	1.03	1.16	2–4	0.86	0.8	0.91	4–7	2.29	2.12	2.45	1	6.48	6.22	6.75	1
	NO (μg/m3)	2.3	0.41	4.22	2–4	1.67	0.99	2.35	3–5	1.12	0.38	1.87	2–4	0.51	− 0.65	1.68	3–7	1.1	0.59	1.61	0*
	NO_2_ (μg/m^3^)	− 1.02	− 2.73	0.72	2–4	4.46	3.41	5.53	0–1	A				5.22	3.97	16.48	7–9	1.9	0.96	2.86	8*
	O_3_ (μg/m^3^)	− 2.23	− 3.1	− 1.36	8	0.54	− 0.55	1.64	0	A				A				− 2.54	− 3.52	− 1.54	4–7*
	SO_2_ (μg/m^3^)	− 0.1	− 4.74	4.76	0	5.54	4	7.11	1–3	0.47	− 0.41	1.35	0–1	4.77	2.13	7.47	1–3	2.83	1.22	4.46	2–3 and 9*
Time series (LR and Almon)	PM2.5 (μg/m^3^)	2.40%	1.90%	2.90%	5–6	A				3.10%	2.29%	3.91%	5	0.80%	0.57%	1.03%	2	3.40%	2.77%	4.03%	7
	PM10 (μg/m^3^)	1.00%	0.70%	1.30%	5	1.10%	0.83%	1.37%	6	0.10%	− 0.03%	0.23%	3	0.90%	0.69%	1.11%	3	1.60%	1.20%	2.00%	7
	PM2.5_24 (μg/m^3^)	4.50%	3.50%	5.50%	6–7	1.90%	1.28%	2.52%	5	3.60%	2.54%	4.66%	7	1.40%	0.94%	1.86%	4	4.50%	3.64%	5.36%	7
	PM10_24 (μg/m^3^)	2.80%	1.73%	3.87%	5–6	1.70%	1.15%	2.25%	5–6	3.10%	2.29%	3.91%	7	1.40%	1.01%	1.79%	3	3.50%	2.77%	4.23%	7
	NO_x_ (μg/m^3^)	1.30%	0.86%	1.74%	4	0.70%	0.45%	0.95%	5–6	0.30%	0.15%	0.45%	3	0.30%	0.22%	0.38%	2	0.50%	0.36%	0.64%	5–6
	NO (μg/m3)	1.90%	1.02%	2.78%	4	1.40%	0.90%	1.90%	6	1.00%	0.60%	1.40%	6	0.20%	0.14%	0.26%	2	0.80%	0.54%	1.06%	6
	NO_2_ (μg/m^3^)	2.90%	1.82%	3.98%	4	3.50%	2.67%	4.33%	4	A				2.60%	2.00%	3.20%	1–2	1.30%	0.76%	1.84%	4
	O_3_ (μg/m^3^)	− 1.70%	− 2.29%	− 1.11%	9	− 2.10%	− 2.78%	− 1.42%	10	A				A				− 1.60%	− 2.12%	− 1.08%	9
	SO_2_ (μg/m^3^)	12.70%	9.52%	15.88%	0	5.40%	4.22%	6.58%	5–6	0.30%	− 0.19%	0.79%	3	7.50%	5.62%	9.38%	3	3.00%	1.97%	4.03%	8

*flat curve, A - Data not available

**Fig. 3:**
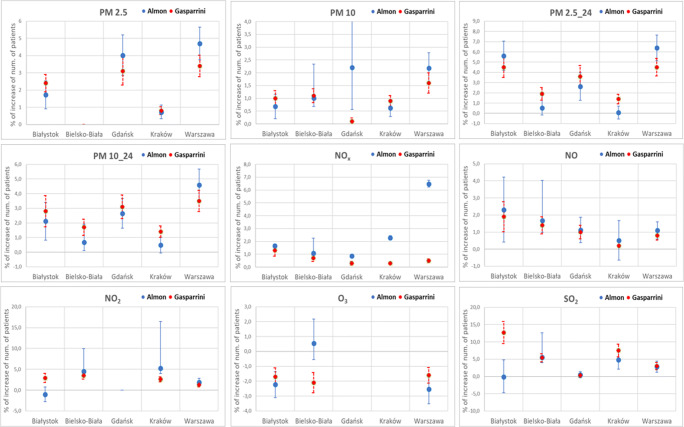
Comparison of percentage increase in hospitalizations for respiratory disease (analysis time-series vs. case-crossover design)

In Fig. 3, the same results are displayed in graphical form, to ease the comparison of the two datasets.

Thirty-five values out of the total 40 show that the calculated % increases of hospitalizations from the two different methods overlap within their respective CI range or show a maximum of 0.5% difference between their respective CI minimums/maximums. As a descriptive example, for Bielsko-Biala, this happens for PM_10_ 24, PM_2.5_ 24, PM_10_, NO_x_, NO, NO_2_, O_3_, and SO_2_. In other words, 88% of the calculated values with both methods display a matching qualitative trend and a quantitative result displaying a CI overlap within a maximum of 0.5% difference in the hospitalization values.

The only major discrepancies of the results have been found in Warsaw (for PM_2.5 24_ and NO_x_), in Krakow (NO_x_), in Biełsko-Biala (O_3_), and in Białystok (SO_2_).

**Fig. 4:**
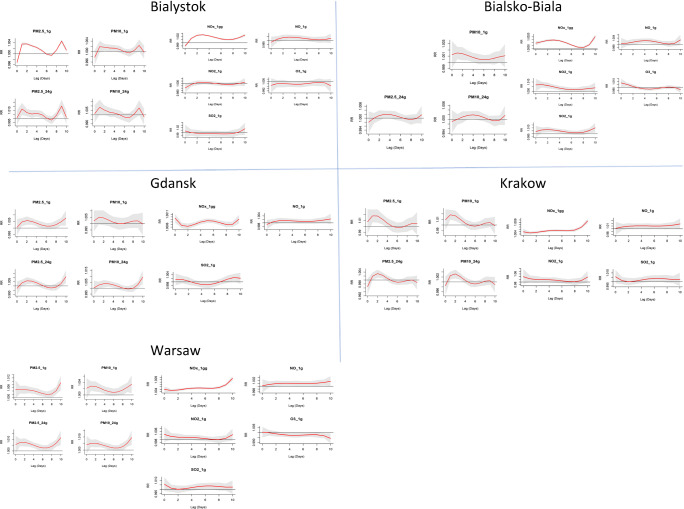
Lag-specific relative risks (with 95% confidence interval) for respiratory hospitalizations associated with a 10-μg/m^3^ increase in air pollutant concentrations. The solid red line represents the predicted relative risk, and dashed lines indicate 95% confidence intervals

The relative risk (RR) plots, needed to analyze the lag effect for all pollutants and cities, are likewise reported in Fig. 4. The maxima in these graphs represent the highest RR for the event to occur, i.e., the highest probability at which the hospitalization increase is expected to happen.

**Fig. 5:**
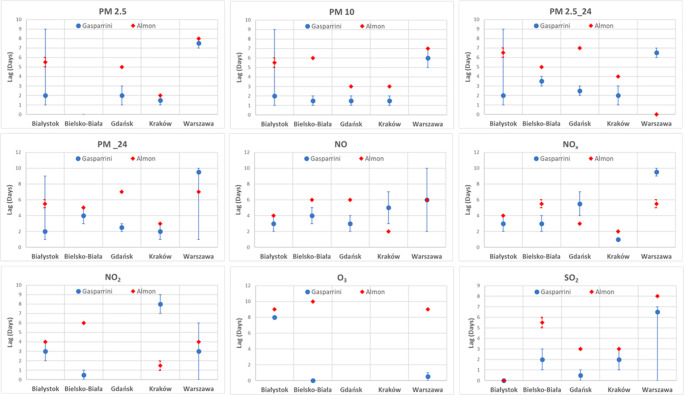
Comparison of calculated maximum lag-specific relative risks for respiratory hospitalizations associated with a 10-μg/m^3^ increase in air pollutant concentrations or both the case-crossover (Gasparrini) and time-series (Almon) analysis

In Fig. 5, the results of the calculated lag where the maximum effect of increased hospitalizations is expected are reported for both case-crossover (Gasparrini DLNM) and time-series (Almon) analysis. From Fig. 5, for 28 out of total 40 calculated lags, the results (RR maxima, i.e., maximum probability of hospitalization after the pollution peak) do not differ for more than 1–2 days between the two methods.

## Discussion

Poland is facing, together with north of Italy and large metropolis (Fusco et al. 2001), some of the highest EU28 levels of air pollution exposures (EEA 2019; Badyda et al. 2016). The main sources of air pollution in Poland originate from exhausts from vehicles, heating (both coal and oil), coal-fired energy production, and industrial emissions. The findings of our previous study and the findings of this analysis both show that the daily average concentrations of the major air pollutants in these five polish cities, object of the analysis, were higher in the winter period and lower in the summer period (with exception of O_3_, which showed an inverse seasonality) and were often well above the Air Quality Standards set forth by the EU legislative frameworks.

Day of the week and temperature was shown in the previous time-series study to have an important effect on the hospitalizations and to remove the bias a normalization of the patients has been performed in the data sample preparation within that analysis. A challenge while performing case-crossover studies using air pollution data is trends and seasonality in the air pollution over time. Case-crossover design is based on the assumption that the exposure distribution is stationary (Peng and Dominici 2008), which is not the case in air pollution levels which fluctuate over time and have distinct trends and seasonal patterns. By matching the day of the week and season, case-crossover study allows control for these confounding by design. The year, month, and holidays as non-overlapping strata have been used to help avoid the bias due to overlap (Taj et al. 2017), matching the days of the week as control days.

Very few studies have been comparing these two methods in general epidemiologic air pollution studies on the same data sample and in particular in the respiratory illness field (Lin et al. 2002; Fung et al. 2003; Hajat 2003; Yuming et al. 2010; Kayo et al. 2009; Tong et al. 2012; Meng et al. 2017). In addition, we did not find so far any published study for which respiratory diseases hospitalizations and air pollution associations have been compared using these two study methodologies.

The difference between the two study results is summarized in Fig. 3, Taking into consideration the very highly different statistical methodologies, these results are encouraging. To a large extent, this could be probably the result of the relatively large number of data points constituting the sample (almost 4 years of hospitalization data). The few studies that did compare these two methodologies, although with different endpoints (e.g., mortality – Meng et al. 2017; Yuming et al. 2010) and not hospitalizations, reported a somehow similar range. In the Meng study (NB with a different end-point, i.e., mortality and not hospitalization), a “10 μg/m^3^ increment of SO_2_ levels was associated with 2.4% and 1.9% increase of relative risk of overall respiratory disease mortality in case-crossover and time-series analyses, respectively”, i.e., a difference of 0.5%. Kayo et al. (2009) (also looking at the mortality endpoint) reported results differing between the methods from 0.53 to 0.88% (i.e., 0.4%), and Tong et al. (2012) reported also differences between the methods in similar ranges, although the endpoint in this latter case was mortality with respect to heatwaves, so a profoundly different setting. In this paper, 88% of the calculated values show overlap of their respective CI’s by a maximum of 0.5%.

The main difference between the results of the two study methodologies seems to be related to the output of the relative magnitude of the CI’s. With the case-crossover design, the CI’s are generally larger. This is coherent with the same findings of the Meng study, where the confidence interval of the time-series analysis was narrower than the one in the case-crossover design and also to earlier literature (Guo et al. 2010).

Where the two study methodologies differed slightly more is in the identification of the maximum lag effect as seen in Table 1.

The distributed lag structure in hospitalizations for respiratory illness has shown to vary largely from maximum effects being recorded at lag = 0, to maximum effects recorded at lags as long as 10–14 days (Kim et al. 2012; Lall et al. 2010; Pun et al. 2015; Tao et al. 2014). The differences in such results have been explained with the typology of the pollutant, the sub-type of respiratory illness, and the stratification of the patient population (kids, elderly, etc.), but there does not seem to be a homogeneous pattern of lag effect predictability.

Like for the % increase in hospitalization, where the data arising from the 2 different methodologies where overlapping at a great extent, also for the lag at which the highest RR (Relative Risk) is displayed, 70% (28 out of 40) of the calculated data points result within 1–2 maximum lag day difference. Plotting the RR utilizing the case-crossover “Gasparrini” design resulted in an easier identification of the lags and a more intuitive understanding of the entity of the effect.

### Study limitations and further research opportunities

The study limitations for the time-series analysis have been discussed in our earlier publication. Common to both methodologies and typical in similar studies in this research area, the data on ambient air pollutant concentrations and meteorological conditions were obtained by databases that utilize mainly fixed monitoring stations. The assumption is that these values represent appropriately the mean exposure for the population, which obviously leads to a measurement error as not all individuals are exposed to the same pollution level. The two study designs have different biases, and the analysis of both together offers the opportunity to look at the same dataset with two different methodologies and hence potentially reduce or test the bias. Typically, the case-crossover design assumes a step-like seasonal change (Zanobetti et al. 2003), while time-series studies assumes a smooth change in the seasonal pattern. Consistent results have been published using both the time-series and the case case-crossover analysis, which supports that both statistical analyses can be performed to estimate air pollution and hospitalization patterns producing robust and comparable results. Further analysis would be beneficial in general to understand better the lag effect structures and perhaps a third statistical method, perhaps using neural networks, could be used to further evaluate the data set. In addition, a further analysis on a lag window longer than 10 days could be performed, also in the light of new literature findings that show that in some cases there can be short time health effects recorded at longer lag intervals (Heresh et al. 2019).

## Conclusions

These analyses show a positive association between air pollutants and hospitalizations for respiratory disease in 5 cities in Poland during the years 2014–2017. The case-crossover design study confirms the previous time-series findings on the same dataset, corroborating the finding that both methodologies are good for studies where case-related health effects are being investigated. By comparing the two methodologies, we found the time series to have narrower CIs. Also for the lag effect, the time-series analysis has shown a narrower time window between the lowest and highest potential lag.
